# Development, Diversity, and Death of MGE-Derived Cortical Interneurons

**DOI:** 10.3390/ijms22179297

**Published:** 2021-08-27

**Authors:** Rhîannan H. Williams, Therese Riedemann

**Affiliations:** 1 Helmholtz Zentrum München, German Research Centre for Environmental Health, Institute for Neurogenomics, Ingolstädter Landstraße 1, 85764 Neuherberg, Germany; rhiannan.williams@helmholtz-muenchen.de; 2 Ludwig-Maximilians-Universität München, Biomedical Center, Physiological Genomics, Grosshaderner Str. 9, 82152 Planegg-Martinsried, Germany

**Keywords:** GABA, cortical interneurons, somatostatin, parvalbumin, interneuron diversity, interneuron development

## Abstract

In the mammalian brain, cortical interneurons (INs) are a highly diverse group of cells. A key neurophysiological question concerns how each class of INs contributes to cortical circuit function and whether specific roles can be attributed to a selective cell type. To address this question, researchers are integrating knowledge derived from transcriptomic, histological, electrophysiological, developmental, and functional experiments to extensively characterise the different classes of INs. Our hope is that such knowledge permits the selective targeting of cell types for therapeutic endeavours. This review will focus on two of the main types of INs, namely the parvalbumin (PV^+^) or somatostatin (SOM^+^)-containing cells, and summarise the research to date on these classes.

## 1. Introduction

The cortex of the mammalian brain is composed of two main neuronal groups: projection neurons and interneurons (INs) [1,2,3,4,5,6,7,8]. Projection neurons are cells whose axons extend from the region where they are located to other brain areas and/or to the spinal cord. In doing so, projection neurons facilitate communication between diverse areas of the central nervous system. In the neocortex, this communication is attributed to pyramidal cells and is virtually always activating, i.e., most often projection neurons excite postsynaptic neurons by releasing the neurotransmitter glutamate. INs on the other hand are classically defined by the fact that their axons reside locally, hence their original name ‘short axon cell’ as described by Ramon y Cajal. The population of cortical INs is highly diverse and composed of different classes of subtypes. INs can be either excitatory or inhibitory depending on the neurotransmitters released. Some interneuron (IN) types are cholinergic or monoaminergic but the vast majority of INs release the neurotransmitter γ-animo butyric acid (GABA), causing the activation of ionotropic or metabotropic GABA receptors. Activation of these pre or postsynaptic GABA receptors results in an increase in the chloride or potassium conductance, leading to a hyperpolarization of the cell and electrical silencing. Together, projection neurons enable global communication in the brain while cortical INs modulate this information flow. This modulation alters the strength of the message via different circuit motifs such as lateral inhibition, feedback, or feedforward inhibition/disinhibition. By directly modulating the excitability of pyramidal neurons, it is not surprising that dysfunctions in the GABAergic system and/or of GABAergic INs result in serious neurological and/or neuropsychiatric defects in human patients. As INs are composed of distinct cell classes, it is necessary to disentangle how the different IN classes assemble to control the correct dynamics of circuit excitability and to understand whether certain INs are more prone to disease than others. To date, it has been documented that different IN types display distinct innervation patterns onto pyramidal neurons to differentially affect their excitability. Moreover, most INs co-release neuropeptides when sufficiently activated, such as the vasoactive intestinal peptide, neuropeptide Y, or somatostatin, resulting in a sustained modulation of pyramidal cell output [9,10,11]. Due to this complexity, the mechanisms for an accurate assembly of neuron types within a given circuit to preserve its function requires both correct proportional expression of cell types and integrated synaptic compatibility. Therefore, the generation, maturation, and refinement of both main neuronal classes, as well as their respective subtypes, is a highly orchestrated developmental process. This review discusses the different INs classification schemes with a focus on parvalbumin (PV^+^) or somatostatin (SOM^+^)-containing INs, discussing the developmental ontology and selective features of these specific INs.

## 2. Interneuron Classification Schemes

### 2.1. Morphology

**Figure 1:**
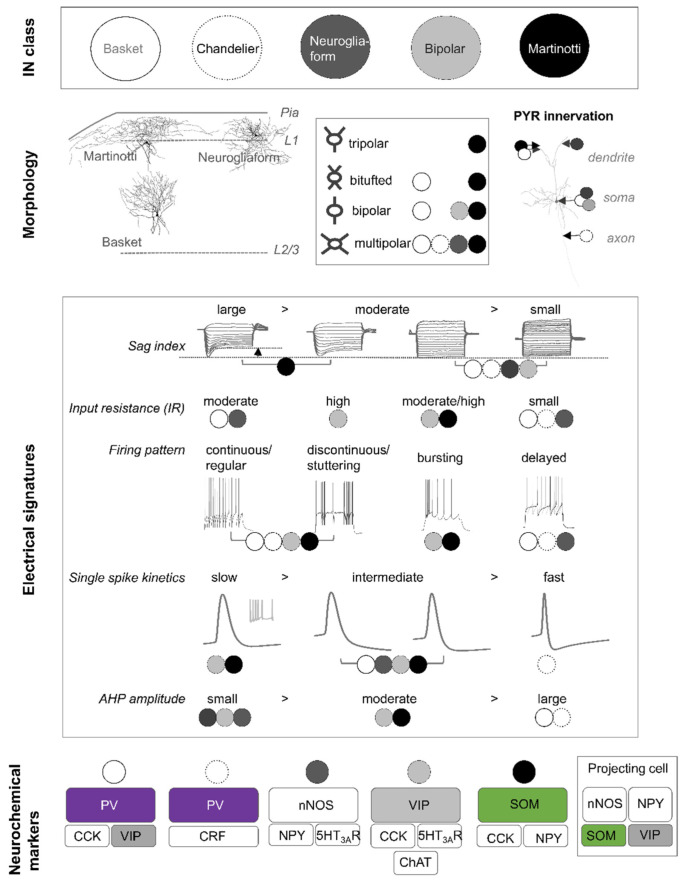
Overview of the major morphological interneuron types in the neocortex. Top: illustration of major morphological IN classes. Upper middle, left: representative reconstructions of Martinotti, neurogliaform, and basket cell type in the neocortex; middle: correlation of somatodendritic to morphological type; and right: schematic representation of preferred postsynaptic target of basket, Martinotti, neurogliaform, and bipolar cells on pyramidal cells (PYR). Lower middle: Schematic illustration of the electrical signatures of these four morphological IN types. Slow single spike kinetics are only observed in burst-spiking neurons. Bottom: expressed neurochemical markers in these IN subtypes. Abbreviations: AHP, afterhyperpolarisation; CCK, cholecystokinin; ChAT, choline acetyltransferase; L, cortical layer; nNOS, neuronal nitric oxide synthase; NPY, neuropeptide Y; PV, parvalbumin; PYR, pyramidal cell; SOM, somatostatin; VIP, vasoactive intestinal peptide; and 5HT_3A_R, 5HT_3A_ receptor.

Traditionally, cortical INs have been characterised by a variety of somatodendritic morphologies (bipolar, multipolar, tufted, and bi-tufted) [12]. An expanded categorisation also considers the axonal ramifications and the synaptic innervation pattern of individual cells. According to the morphological classification scheme, 10 different cortical IN types have been distinguished, although several subtypes exist [13]. Some of the best studied morphological cell types in the rodent neocortex represent basket cells [14,15,16,17,18,19,20], chandelier cells [21,22,23], bipolar cells [12,24,25], neurogliaform cells [26,27,28], and Martinotti cells [25,29,30,31,32] (Figure 1). Most of these data are either derived from electron microscopy studies or from visualisation and reconstruction of patched cells in brain slices following electrophysiological recordings. An example of different morphological cell types found in the anterior cingulate mouse cortex is depicted in Figure 1. The axons of basket cells predominately reside within their home layer and typically form perisomatic ‘basket’ terminals with the soma and proximal dendrites of neighbouring cells. Basket cells classically make depressing synapses onto pyramidal neurons [33,34,35]. In contrast, axons from Martinotti cells spread across layers and ramify extensively in layer 1 (Figure 1). The preferred postsynaptic target of Martinotti cells, whereby they facilitate synapses [33,34,35], are distal dendrites of neighbouring pyramidal neurons. In addition, Martinotti cells also inhibit other INs. Neurogliaform cells are best characterised by their relatively small multipolar soma and dendritic tree (Figure 1), and by their remarkably high presynaptic bouton density [28,36]. As some of their presynaptic boutons are not associated with postsynaptic structures, it is suggested that these INs contribute to GABA volume transmission in the neocortex [37]. Neurogliaform cells inhibit all neurons within their sphere of influence, without any preference towards a specific cell type [38,39]. In summary, the morphology of INs reflects known functions of these cell types and permits the identification of presynaptic inputs and postsynaptic outputs. Nevertheless, morphological reconstructions from patched cells in brain slices are not foolproof and full reconstructions of complete neurite trees may not be possible because of the slice thickness or individual cell orientation in a slice. To overcome this limitation, great progress is being made using viral strategies in the living animal as well as through advanced microscopy techniques [1]. Sparse viral labelling of projection neurons in the cortex together with serial two-photon microscopy of optically cleared brain tissue allows for the near to complete visualisation and subsequent reconstruction of neuronal projections in the entire brain [1] and promotes our understanding of the connectivity of identified neurons. To date, morphological classifications represent a strong and valid experimental approach to defining INs classes, yet these classes are continuously reevaluated and redefined when combined with ultrastructural, electrophysiological, neurochemical, and/or transcriptomic data.

### 2.2. Electrophysiology

Historically, cell classes were derived based on three main electrophysiological features: (1) action potential discharge pattern, (2) single spike kinetics, and (3) passive membrane properties (resting membrane potential, input resistance, and cell capacitance). Based on firing patterns, all cortical INs can be assigned to one of four parent categories with each consisting of multiple subordinate groups. The parent categories distinguish cortical INs that respond to a suprathreshold depolarising current pulse with (1) continuous (or regular), (2) discontinuous (or stuttering), (3) burst-spiking, or (4) a delayed action potential discharge behaviour (Figure 1). The subordinate categories further distinguish between adapting, accommodating, irregular spiking, or accelerating IN types [13]. Furthermore, some INs are characterised by a low-threshold spike [40]. In contrast to the classification scheme based on morphological data, electrophysiological classification is associated with a high degree of variability and most morphological types show diverse action potential firing patterns. Nonetheless, a delayed action potential discharge pattern upon just suprathreshold current injection is typically associated with neurogliaform cells. Unfortunately, a similar correlation is not possible for most other IN types: however, basket cells and chandelier cells can best be characterised by their fast single spike kinetics and low input resistances (Figure 1). In contrast, Martinotti and bipolar cells exhibit intermediate single spike kinetics and moderate to high input resistances. In addition, analysis of the current–voltage relationship in morphological IN types reveals that large sag indices are typically observed in Martinotti cells. Therefore, the combined analysis of firing patterns [41] with other electrophysiological features such as intrinsic membrane properties, current–voltage relationships, single spike kinetics, and afterhyperpolarisation properties helps to sharpen the segregation of specific IN subtypes. Ideally, analysis of these properties should be combined with an analysis of pre and postsynaptic assessment for each morphological cell type.

### 2.3. Neurochemistry

GABAergic INs are currently grouped into three major classes according to their neurochemical expression profiles [42]. These classes are virtually non-overlapping and correspond to INs positive for the (1) parvalbumin (PV^+^), (2) somatostatin (SOM^+^), and (3) 5-HT_3A_ receptor (5-HT_3A_R^+^). 5-HT_3A_R^+^ INs can in turn be divided into cells expressing either vasoactive intestinal peptide (VIP^+^) or Reelin (Reln, non-VIP INs) [43,44]. Moreover, despite these main classes, many INs coexpress a combination of different markers (including calcium-binding proteins, neuropeptides, and neurotransmitter receptors and enzymes), multiplying the number of neurochemical subtypes (Figure 1). As most INs belonging to one of the three neurochemical classes exhibit clear differences regarding electrophysiological and/or synaptic output properties, this neurochemical categorisation is a well-established and respected experimental approach to study IN function in specific cell types.

### 2.4. Transcriptomics

The availability of single-cell sequencing together with publicly available sequencing databases (e.g., https://portal.brain-map.org/atlases-and-data/rnaseq) has significantly increased our understanding of cortical INs by enabling an in-depth analysis of individual neurons to evolve the traditional classification schemes [45,46,47,48]. According to transcriptomic profiling, cortical INs are clustered into 10 distinct classes and can further be sub-classified into more than 20 different subtypes. These distinct classes are characterised by the differential expression of neuropeptides, transcription factors, ion channels, neurotransmitter receptors, or calcium-binding proteins, among others. Interestingly, the designation of many IN classes mirrors their specific embryonic origin. However, it remains to be tested whether each transcriptomic subtype translates into a functional class within a given cortical circuit [49,50]. At present, there is not always full accordance between morphological, electrophysiological, and transcriptomic classifications, although in most cases there are good agreements [45,46,51,52]. While transcriptomic profiling is still in its scientific infancy compared to either classical morphological or electrophysiological approaches, it is apparent that an updated taxonomy of cortical interneuron subtypes is required.

## 3. Laminar Distribution of Cortical Interneurons

**Figure 2:**
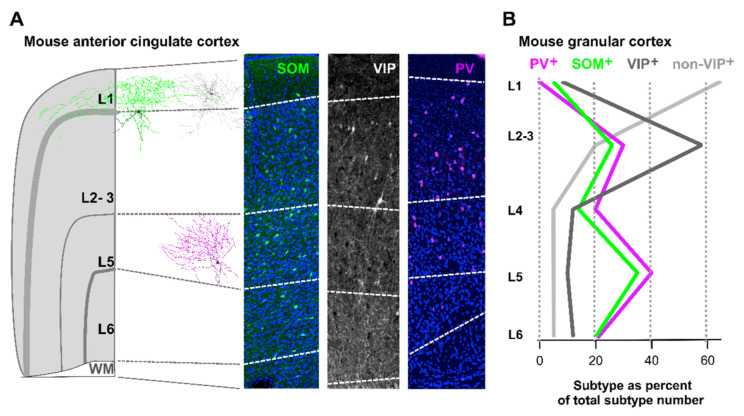
Laminar distribution profile of PV^+^, SOM^+^, VIP^+^, and non-VIP INs in the neocortex. (**A**) Left panel: schematic illustration of the mouse anterior cingulate cortex containing 1 Martinotti cell (green), 1 neurogliaform cell (grey), and 1 basket cell (magenta). Right Panel: confocal images (maximum intensity projections) of coronal sections of the mouse anterior cingulate cortex labelled for SOM^+^ (left, green), VIP^+^ (middle, white), and PV^+^ (left, magenta) neurons. Nuclei were visualised with DAPI (blue) to identify cortical layers (left and right). (**B**) Diagram showing the relative fraction of PV^+^ (magenta), SOM^+^ (green), VIP^+^ (dark grey), and non-VIP (light grey) INs as function of the cortical layer. PV and SOM expression can be observed in supra (L2/3) and infragranular (L5/6) layers, whereas that of VIP is restricted to supragranular layers.

In the adult neocortex, PV^+^ INs are the most frequent, comprising between 40–50% of all INs. The two other types, SOM^+^ and 5-HT_3A_R^+^ (VIP^+^ and non-VIP) INs, each contribute approximately 20–30% to the remaining proportion [42]. However, the relative proportions of each IN type to the overall population of GABAergic INs exhibit region-specific differences: The relative density of PV^+^ INs tends to be higher in the motor and somatosensory cortex, whereas for VIP^+^ INs, the highest density is in the visual cortex [53]. In addition, each IN class has a unique laminar distribution pattern. VIP^+^ INs are preferably located in supragranular cortical layers [54,55,56,57]. Similarly, non-VIP INs, most of which are Reln^+^, NPY^+^, and/or nNOS^+^ INs, are almost exclusively located in the superficial layers of the neocortex [58]. In contrast, the expression of PV^+^ and SOM^+^ INs tends to be greatest in layer 5. INs present in cortical layer 1 are typically of the non-VIP type but a small fraction of VIP^+^ and SOM^+^ INs can also be observed in layer 1. SOM^+^ INs present in layer 1 are preferably located at the border to layer 2. No PV^+^ INs are found in layer 1 (Figure 2).

## 4. Origin and Development of PV^+^ and SOM^+^ Interneurons

**Figure 3:**
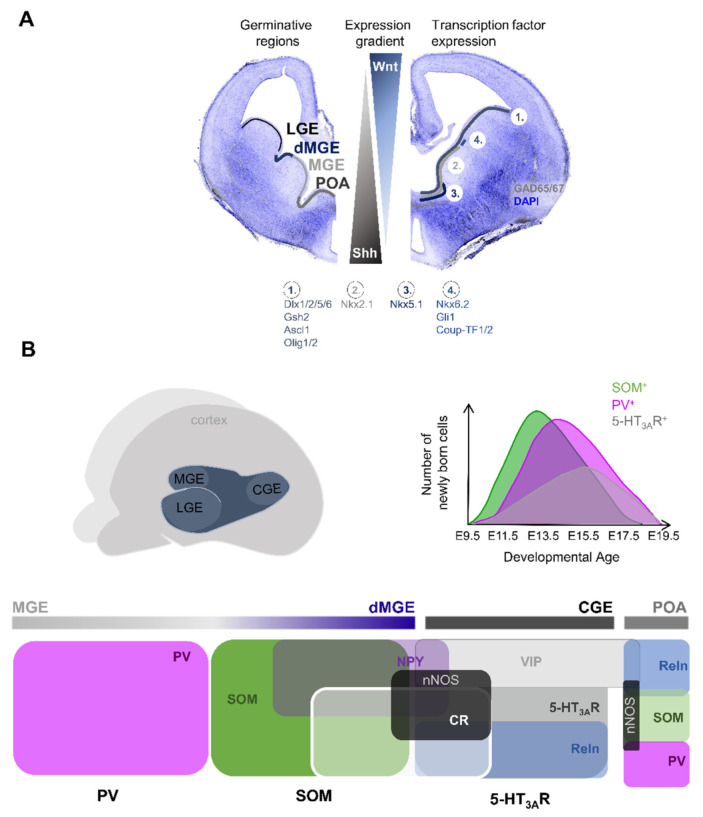
Embryonic origin and development of cortical interneurons. (**A**) Confocal image of a E14.5 coronal brain slice stained for GAD65/67 (grey) and DAPI (blue). The germinative regions (LGE, dMGE, MGE, and POA) are illustrated on the left hemisphere. The expression of distinct transcription factors is illustrated on the right hemisphere. The graded expression of Shh and Wnt is depicted in the middle. (**B**) Top, left panel: schematic representation of the brain with germinative regions illustrated in dark blue; and right panel: diagram showing the generation of SOM^+^, PV^+^, and 5-HT3AR^+^ INs as a function of developmental age. Bottom: schematic of germinative regions in the embryonic brain and their relative contribution to classes of INs. The MGE gives preferentially rise to PV^+^ and SOM^+^ INs, whereas the CGE primarily produces 5-HT_3A_R^+^ INs. The POA in turn produces a mixed population of GABAergic INs. MGE and CGE-derived INs typically coexpress a combination of different neurochemical markers, some of which are illustrated.

Around 90% of all cortical INs are derived from the ganglionic eminences (GE); 60% are derived from the medial GE (MGE) and 30% are born in the caudal GE (CGE). The preoptic area (POA) produces the remaining 10% of cortical INs (Figure 3).

The germinative regions are divided into a ventricular zone (VZ) and a subventricular zone (SVZ), each hosting distinct neural precursors or progenitors. During early stages of cortical IN neurogenesis, most progenitor divisions occur at the ventricular surface. The SVZ develops at around E11 and progressively expands over time. By around E13–E14, the SVZ is the main site of progenitor proliferation and is composed of a great variety of progenitor types [59]. Electrophysiological recordings of neural progenitor cells and radial glial cells within the VZ showed that these cells are connected electrically via gap junctions well before synapse formation [60,61,62,63,64]. The number of electrically coupled cells becomes progressively smaller at later stages of development [60], suggesting that electrical synapses play a crucial role during progenitor proliferation [65] by coupling electrical activity to cell cycle transition. In addition, in vitro studies support the idea that neuronal or oligodendrocyte lineage is at least partly established by gap junctional coupling [66,67]. To date, it is unclear whether electrical coupling also affects IN subtype specification.

SOM^+^ INs are thought to be generated from short neural precursors within the VZ of MGE. These MGE-derived SOM^+^ INs and POA-derived INs represent the earliest born INs [23,68,69]. PV^+^ INs are generated by intermediate progenitors of the MGE SVZ [70] and are the next born INs, finally followed by CGE-derived INs [23,68,69] (Figure 3B).

### 4.1. Morphogens and Cell Specificity

Many studies suggest that pattern formation and fate specification of cortical INs is, at least partly, accomplished in the proliferative zones of the embryonic brain. Two hypotheses exist: (1) the generation of ‘specific progenitors’ in the VZ that give rise to specific IN subtypes or (2) the generation of ‘cardinal progenitors’ with a fluid identity [44,71]. The generation of these ‘specific progenitors’ requires the existence of local and chronological gradients of certain signalling molecules (‘morphogens’) and transcription factors that induce a specific cell fate in these progenitor cells. Such morphogens exhibit a topographical bias along the dorso-ventral or rostro-caudal axis of the GE [72,73,74], establishing a graded expression of downstream signalling molecules and transcription factors. There are four main protein morphogen families: Wnts, Sonic hedgehog (Shh), bone morphogenetic proteins (BMP), and Fibroblast growth factors (FGF).

Morphogens such as BMP or Wnts are secreted from the cortical hem while Sonic hedgehog (Shh) is secreted from the floor plate (Figure 3A). The location of the cortical hem adjacent to the MGE is such that BMP and Wnt secretion establishes a caudomedial (high) to ventrolateral (low) gradient along the MGE. Shh secretion from the floor plate results in a ventro-dorsal gradient. Conditional knockout animals, homotopic transplantation, and pharmacologic interventions have been instrumental in demonstrating the role of such morphogens in the fate specification of cortical INs [75,76,77,78]. Specifically, loss of Wnt signalling during embryonic development promotes a PV^+^ phenotype and transplantation of labelled rostral (low Wnt levels) MGE cells at E12.5 into E13.5 unlabelled host embryos results in a strong bias towards PV^+^ INs in the neocortex [75].

FGF promotes ventral and anterior telencephalic fates and indirectly inhibits BMP activity [79,80]. During embryonic brain development, FGF is secreted by the anterior forebrain and establishes a rostro-caudal and ventro-dorsal gradient to assist the specific graded expression patterns of transcription factors that result in the development of IN subtypes. Shh also acts as a positive feedforward molecule to drive secondary Shh signalling in the forebrain [81], in which it helps to maintain regional identity by inducing the expression of the homeobox transcription factor Nk2 homeobox (Nkx2.1) in the pallidal proliferative zone [76].

### 4.2. Transcription Factors and Establishment of Regional Identity

Transcription factors importantly contribute to cell specification and differentiation to generate cortical INs. Transcription factors mediating GABAergic IN fate are Ascl1, Dlx1/2, Dlx5/6, Gsh2, and Olig2 [82,83,84,85,86,87]. Regional identity (e.g., MGE vs. POA-derived INs) and subtype specification is then achieved by the localised expression of transcription factors or combinations thereof. There have been several key transcription factors identified: two of which are Nkx2.1 and Nkx6.2, produced from the *Nk2* homeobox, whereas Nkx2.1 is expressed in the MGE and POA, and Nkx6.2 expression is limited to the dorsal aspect of the MGE [88,89] (Figure 3A). Both are involved in shaping the cell types derived from the MGE. Nkx2.1 shares reciprocity with Shh and when active, maintains Shh expression [90,91]. It is involved in modifying regulatory elements to sculpt the identity of the resulting GABAergic INs from progenitors and specify regional identity. For example, Nkx2.1 loss-of-function studies in cells promoted the generation of LGE and CGE cell types at the expense of MGE-derived cells (e.g., cholinergic pallidal projection neurons), indicating a shift in the fate caused by altered lineage and region-specific genes [92,93,94] and suggesting that Nkx2.1 represses progenitor domains adjacent to the MGE [71]. Overall, Nkx2.1 acts as a master regulator of MGE progenitor identity. In comparison, Nkx6.2 preferentially regulates cell differentiation in precursors rather than in proliferating progenitors [88].

Additionally, Nkx2.1 affects cell specificity via the upstream induction of Lhx6 and Lhx8 Lim-homeobox genes responsible for the development of the globus pallidus [95]. LIM-homeobox genes are necessary for Lhx6 expression and together they enrich activating regulator elements to promote transcriptional activation and differentiation of MGE-derived INs [95]. Lhx6 is then sufficient to drive the expression of Arx (aristaless-related homeobox) and the chemokine receptor 7 (Cxcr7) to generate PV^+^ or SOM^+^ INs [96,97,98,99]. Disruption of this Lhx6 pathway, such as via conditional knockout of the transcription factor Sp9 during embryonic development, results in significantly reduced cortical PV^+^ and SOM^+^ IN numbers in the adult (P30) cortex.

Furthermore, the homeodomain transcription factor orthodenticle 2 (OTX2) is required for a rostro-ventral MGE identity and OTX2 knockout shifts the fate of the MGE-derived cells towards POA-derived cells [100].

The expression of transcription factors CoupTF-1 and CoupTF-2 is restricted to a continuous rostro-dorsal arc within the MGE VZ and the expression of both transcription factors becomes gradually limited to the dorsal MGE. In addition, CoupTF-1/2 is highly enriched in the CGE. Accordingly, Coup-TF1/2 is implicated in preferentially generating SOM^+^ and CGE-derived cortical INs (Figure 3A) [101,102,103].

Similar to the limited expression of Nkx6.2 in the dorsal MGE, the expression of the Shh-responsive gene Gli1 is also restricted to the dorsal MGE and promotes the generation of SOM^+^ INs [104,105].

Given the graded expression of morphogens and transcription factors or combinations thereof, the germinative regions of the ganglionic eminences and of the preoptic area obtain regional identity and mostly produce distinct IN types. The MGE mainly gives rise to PV^+^ and SOM^+^ INs, whereas the CGE generates the majority of 5-HT_3A_R^+^ INs (Figure 3B). In contrast, the POA produces a diverse group of cortical INs comprising, amongst others, PV^+^, SOM^+^, and VIP^+^ INs [68] (Figure 3B). Furthermore, transplantation of fluorescently labelled dMGE precursors resulted in grafted cells predominantly adopting a SOM^+^ IN phenotype [106]. This indicated that most SOM^+^ INs are generated from the dorsal aspect of the MGE (dMGE), whereas the MGE produces PV^+^ INs [77]. Fate-mapping studies further revealed that SOM^+^ INs coexpressing CR are preferentially generated in the dMGE, whereas those coexpressing NPY are primarily produced in the MGE [89]. As mentioned earlier, CGE-derived INs can further be subdivided into VIP^+^ or non-VIP Reln^+^ INs, both of which can coexpress nNOS and/or NPY [58,107] (Figure 3B).

It is not yet fully understood whether these different neurochemical IN types observed in the adult cortex are derived from specific progenitors within the germinative zone or whether mature neurochemical IN types are generated from cardinal progenitors. The latter are then sculpted into final specifications in the adult cortex depending on the cortical activity, connectivity, and function of a given IN within the cortical circuit.

### 4.3. Interneuron Diversity from Specific versus Cardinal Progenitors

In favour of the specific progenitor hypothesis is the finding that the generation of cortical INs follows an intrinsic schedule: first SOM^+^ and then PV^+^ INs are born from the MGE, indicating the generation of IN subtypes from specific progenitors [68,70]. This hypothesis is further supported by recent single-cell transcriptomics studies showing that MGE progenitors constitute a highly heterogenous group of cells [59,108,109], arguing for the existence of specific progenitors from which specific adult cortical IN types are derived.

Nevertheless, there appears a grey area whereby there is room for switching cell types between either PV^+^ or SOM^+^ phenotypes from a progenitor. Clonally related MGE progenitors give rise to PV^+^ and SOM^+^ INs [108,110,111,112], and conditional knockout of the tsc1 gene can shift the phenotype of SOM^+^ INs towards a PV^+^ IN phenotype [113]. These data suggest that cardinal progenitors are generated with a fluid identity. Considering that the majority of transcription factors occur in SOM^+^ INs [71], it is postulated that PV^+^ cell identity represents the ‘default state’ of MGE-derived INs and only the active suppression of this state, by induction of select transcription factors, permits a SOM^+^ IN phenotype. The difficulty in simulating the expression of time, location, and tissue-dependent transcription factors in vitro is highlighted by only partial establishment of in vivo PV:SOM IN ratios from mouse embryonic stem cell-derived interneurons when transplanted into the somatosensory cortex [114,115,116].

To fully discern whether single progenitors allow for a fluid differentiation and maturation into different IN subtypes, single-cell spatial transcriptomics should be used [117,118]. This will enable a time-dependent analysis of single-cell specification from the same cell. Such an experiment should be combined with a functional readout to discern the role of transcriptomic subtypes in final cell physiology/morphology categorisation [50].

### 4.4. Migration

Newly generated cortical INs pause in the subventricular zone before migrating into the neocortex. A general rule is that earlier-born INs settle in the deeper cortical layers, while later-born cortical INs invade the more superficial layers. Around 50% of MGE-derived SOM^+^ INs and a subgroup of PV^+^ INs preferentially migrate along the superficial migratory stream [119]. Several migration cues are required to assist in the correct migration path to the neocortex and hence altered expression of these guidance cues is often associated with reduced numbers of cortical GABAergic INs [120,121,122]. In addition, ambient GABA and glutamate levels initially promote IN migration through activation of GABA_A_ and AMPA/NMDA receptors [123,124]. GABA promotes migration by modifying intracellular calcium levels and the expression of the K^+^/Cl^−^ cotransporter KCC2 [123,124,125]. Increased KCC2 levels inhibit IN motility so that the cell stops and matures in its given cortical location. INs that exhibit migratory defects, for example, due to lack of guidance cues and/or antagonised membrane receptors, preferentially undergo programmed cell death, which may ensure the perseveration of correct circuit assembly by physiologically healthy neurons [126].

## 5. Postnatal Programmed Cell Death of MGE-Derived Interneurons

The peak number of cortical GABAergic INs occurs at P5–7 and then cell numbers reduce by around 30% within the next 10–12 days [127,128,129]. At the end of the second postnatal week, cortical IN numbers reach a steady state. These data suggest an active pruning of GABAergic INs to ensure an appropriate configuration is established. This pruning is via programmed cell death apoptosis and follows a strict temporal pattern [130]. A family of evolutionary conserved enzymes accomplishes apoptosis. These enzymes, known as caspases, function as cysteine proteases. Caspases are differentially recruited depending on whether cell death occurs in response to intrinsic or extrinsic factors. Extrinsic cell death is initiated by the activation of death receptors that in turn activate caspase-8. Intrinsic cell death is initiated by activation of caspase-9. Both signalling pathways converge onto the effector caspases-3 and/or caspases-7 that trigger the demise of the cell. For example, when E13.5 MGE-derived cells are grown in culture and then transplanted into the cortex, the peak of cell death occurs 15 days after transplantation, mimicking the timeline of the native in vivo IN cell fate [127].

### Control of Apoptosis

Several signalling molecules can act as promoters or inhibitors of apoptosis. Proapoptotic proteins include the Bcl2-associated X protein (Bax) and Bcl2 antagonist/killer 1 (Bak), while Bcl2 and Blc2-like 1 (Blc-Xl) act as antiapoptotic factors. In the developing cortex, programmed IN cell death is Bax-dependent, is not modulated by neurotrophin signalling, and is modified by cell adhesion molecules of the protocadherin subgroup [127,131]. Interestingly, programmed cell death of inhibitory INs appears directly coupled to reduced excitatory activity and death of excitatory neurons. Indeed, it has been shown that (1) pyramidal cell death occurs before IN cell death; (2) P7 INs have significantly reduced neuronal activity 24 h before their death; and (3) (chemo)genetic pyramidal cell activation between P5 and P8 resulted in a significant increase in cortical PV^+^ and SOM^+^ IN numbers via reduced apoptotic IN events [132,133]. Equally, the rate of IN cell death is matched to that of pyramidal cell numbers to maintain a physiological excitatory-to-inhibitory neuron ratio in the neocortex.

Developmental apoptosis has region and cortical layer-specific differences [134]. More medial (motor cortex M1) superficial cortical layers have the highest numbers of induced cell death versus deep cortical layers and lateral cortical regions (somatosensory cortex S1). Coincidently, the numbers of cortical INs are highest in the somatosensory cortex compared to the motor cortex and are higher in the deeper cortical layers compared to the more superficial cortical layers (mean number of cells/mm^2^) [53].

Intriguingly, premature birth leads to advanced neuronal cell death if cell death rates are compared as a function of post-conception age. Conversely, a delayed birth does not delay the rate of programmed cell death [135]. It is therefore suggested that programmed cell death follows an intrinsic developmental program that can be accelerated by an advanced birth. The underlying mechanisms of advancing programmed cell death are currently not well understood but are likely to be influenced by hormones.

## 6. Postnatal Maturation of SOM^+^ and PV^+^ Interneurons

### 6.1. Onset of PV and SOM Expression in Neocortex

By the first postnatal week, cortical IN migration is almost complete. Subsequently, the migrating distance of GABAergic interneurons is significantly reduced [123,124] and cortical SOM protein expression increases. By the second postnatal week, cortical PV protein expression also occurs, resulting in the recognition of cortical SOM^+^ and PV^+^ INs [120,136,137,138,139,140]. The chronological order of the protein expression of SOM and then PV appears to reflect the embryonic development of these two INs. In addition, there are also brain region-specific chronological expression patterns [54,57]. For example, auditory cortical tracing studies indicate that PV^+^ IN numbers stabilize by around P35, whereas SOM^+^ IN numbers increase until P145 in this region. In contrast, adult levels of SOM^+^ and PV^+^ INs in the visual cortex are already reached after the third postnatal week, suggesting cortex area-specific developmental differences [57]. It is unclear whether the development of specific neurochemical subtypes of PV^+^ and/or SOM^+^ IN types is accomplished by the time that SOM/PV levels reach a steady state in the neocortex or whether subtype differentiation continues until later developmental stages.

### 6.2. Development of Connectivity

Ex vivo electrophysiological recordings of postnatal PV^+^ and SOM^+^ INs demonstrate that both IN types acquire their final passive and active membrane properties after the second postnatal week [141,142,143,144,145,146]. Specifically, steady-state characteristics of passive membrane properties (resting membrane potential, input resistance, and somatic time constant) and maturation of single spike or action potential (AP) discharge properties of SOM^+^ INs occur by P21 [141,142,143]. Similarly, PV^+^ INs adopt their final electrophysiological phenotype by P21-P30 [144,145,146,147,148]. Spontaneous synaptic input onto SOM^+^ INs increases with postnatal development [141,142,144,146]. Paired recordings between pyramidal neurons and SOM^+^ INs indicate increased connectivity strength with age [142,144,149] and dual patch-clamp recordings between pairs of SOM^+^ or pairs of PV^+^ INs show increased electrical and chemical coupling probability with neuronal maturation, with a steady-state accomplished by P30 [141,144,147,150,151].

The synaptic relationship between SOM^+^ or PV^+^ INs onto pyramidal neurons has been best studied in the visual cortex. The former relationship is characterised by three main findings [142,151]: (1) it emerges around P6, (2) the connection probability increases with ongoing maturation, and (3) the strength of synaptic transmission appears linked to the eye-opening period of mice (around P14) [121,142,151]. In contrast, the synaptic connectivity of the latter relationship, once established, remains stable and the synaptic output is strengthened during the time of eye opening [151]. Moreover, the time course of PV^+^ IN maturation and coupling to pyramidal cells is best reflected by the development of gamma oscillations in the neocortex [140]. Interestingly, sensory deprivation and experience affect the maturation of PV^+^ and SOM^+^ INs, and the effects are more pronounced on PV^+^ Ins, suggesting that cortical activity levels, particularly if reduced, may contribute to the maturation of cortical INs and their connectivity [141,152,153,154].

Interneuronal activity levels during early postnatal development assist in the correct circuit assembly: (1) earlier-born SOM^+^ INs within the infragranular layer act as a transient intermediate relay between the thalamus, PV^+^ interneurons, and pyramidal neurons [155]; (2) early-born MGE-derived GABAergic INs, of which many become SOM^+^, act as cortical hub neurons that are able to generate recurrent network bursts [156]; (3) ablation of early-generated INs, 60% of which are SOM^+^, impairs the development of GABAergic synaptic inputs onto layer 5 pyramidal neurons [157]; and (4) SOM^+^ INs play a paracrine role in the assembly of perisomatic inhibitory synapses by expressing Collagen XIX, an extracellular matrix protein that is necessary for perisomatic neuron assembly [158,159]. The specific loss of Collagen XIX in SOM^+^ INs results in a dramatic loss of perisomatic nerve terminals onto pyramidal neurons [160].

To our knowledge, there are no studies investigating age-dependent spine formation in SOM^+^ or PV^+^ INs in the neocortex but this would be an interesting concept as spine formation and spine motility are linked to synaptic activity in both pyramidal and GABAergic INs [161,162,163,164]. In contrast to pyramidal cells, spine formation in SOM^+^ INs and possibly also in PV^+^ INs seems to be influenced by the expression of the polysialylated form of the neural adhesion molecule (PSA-NCAM) [165,166]. Therefore, given that spine formation in pyramidal cells and cortical INs is modulated by synaptic activity, albeit by partly diverging mechanisms, it could be postulated that the developmental profile of spine formation in SOM^+^ and PV^+^ INs mimics that of neocortical pyramidal cells. Accordingly, spine formation in cortical INs appears to be accomplished after electrophysiological maturation. Furthermore, spine formation in pyramidal cells is influenced by IN activity. Specifically, learning-induced spine formation in pyramidal cells of the motor cortex exhibits a higher dependence on SOM^+^ IN activity compared to that of PV^+^ INs and has direct effects on the learning of stereotyped movements. This corroborates the hypothesis that SOM^+^ INs play an active role in the arrangement of the synaptic circuitry [160,167].

While it is well established that activity and experience shape IN maturation, the underlying cellular mechanisms are only partially understood but likely include activity-dependent signalling pathways that ultimately cause differential gene expression in subsets of INs [44]. These signalling pathways may be modulated by activity within the millisecond-to-seconds range but also by diurnal or even seasonal activity [152].

In summary, electrophysiological recordings suggest that once SOM^+^ and PV^+^ INs have migrated to the cortex, their maturation is critically dependent on receiving synaptic input in order to successfully integrate into and modify the neuronal activity within a given cortical circuit.

## 7. Properties of PV^+^ INs

### 7.1. Morphology of PV^+^ INs and Expression of Neurochemical Markers

**Figure 4:**
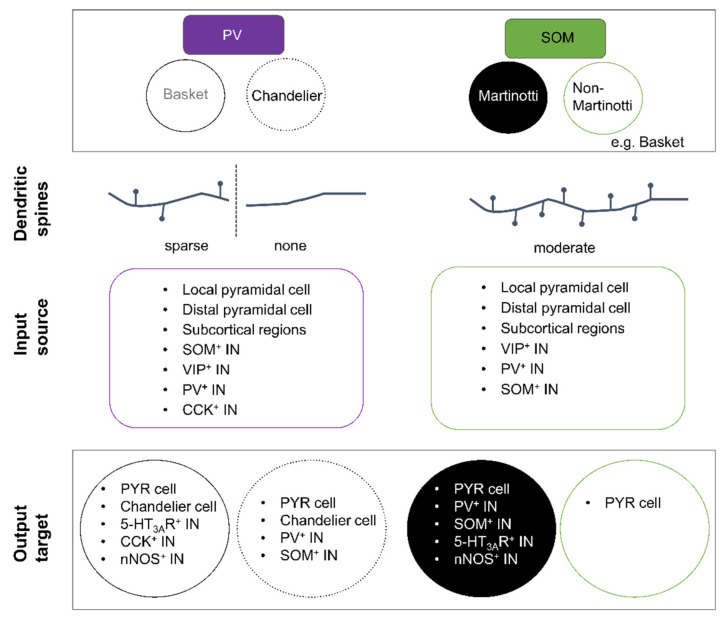
Overview of connectivity (presynaptic inputs and postsynaptic outputs) of neocortical PV^+^ and SOM^+^ INs. PV^+^ INs are subdivided into basket and chandelier cells, and SOM^+^ INs are subclassified into Martinotti and non-Martinotti cells. Dendritic spines can be found on PV^+^ basket cells and on Martinotti and non-Martinotti cells. Dendrites of chandelier cells are always aspiny. The main input source onto any IN type represents pyramidal neurons but PV^+^ and SOM^+^ INs also receive inhibitory inputs from other INs. Main output targets of any IN type are pyramidal cells followed by other IN types.

PV^+^ INs are the most distinguishable IN class with respect to their electrophysiological properties. The majority of PV^+^ cells are either basket or chandelier cells. Both cell types are characterised by a round-to-oval shaped soma with, in general, a multipolar somatodendritic morphology (Figure 1). Most basket cells appear to share electrical and/or chemical synapses with each other [168,169], while chandelier cells appear to only share electrical connections. Initial studies described basket cells as aspiny INs [40,170,171]; however, more recent studies suggest that the dendrites of certain basket cell subtypes are sparsely spiny with a mean spine density of around 1–2 per 10 µm in the neocortex [172,173]. In contrast to basket cells, dendrites of chandelier cells are always aspiny (Figure 4). The axon of chandelier cells forms vertically oriented axon terminals, the so-called cartridges. These cartridges contain a string of presynaptic boutons that specifically innervate the axon initial segment of neighbouring pyramidal neurons, thus providing a powerful modulation of pyramidal cell output [32,174]. Worthy to note, not all chandelier cells express PV and it remains to be tested whether non-PV chandelier cells are functionally distinct [175,176].

Typically, subtypes of PV^+^ INs are known to coexpress the calcium-binding protein calbindin (CB), neuropeptide cholecystokinin (CCK), or glycoprotein reelin (Reln) [6,46,56,177,178]. If of chandelier morphology, DOCK7, which is essential for synaptic development, will be expressed [179,180], as well as the cell adhesion molecule Cadherin-6 [181] and VIP receptor 2 [182].

A small subpopulation of prefrontal PV^+^ neurons are classed as long-range projection neurons as they project out of the cortex. One set innervates the nucleus accumbens and they elicit avoidance behaviour in mice [183]. Other long-range PV^+^ populations in the auditory cortex have been shown to innervate the contralateral auditory cortex [184] or auditory striatum [185]. Interestingly, these long-range PV^+^ neurons display distinct electrophysiological properties that distinguish them from the main class of PV^+^ INs and may reflect a differential role in relaying cortical circuit function [184].

### 7.2. Electrophysiological Properties of PV^+^ INs

PV^+^ INs are often referred to as fast-spiking cells as the majority display fast spike kinetics with a pronounced and fast afterhyperpolarisation. Expression of voltage-gated potassium channels (Kv3.1 subtype) [58,186,187] permits the cells to fire sustainably at high frequencies (>200 Hz), yet PV^+^ INs have diverse firing patterns: The majority exhibit regular, delayed, or stuttering/discontinuous action potential firing. In addition, PV^+^ INs typically display characteristic passive membrane properties that make them less excitable compared to SOM^+^ INs (summarised in Figure 1).

## 8. Properties of SOM^+^ INs

SOM^+^ INs represent a very diverse group of neurons with a myriad of morphological, electrophysiological, and neurochemical properties, resulting in many subtypes [3,4,31,188]. Nevertheless, SOM^+^ INs remain distinguishable from PV^+^ cells. Broadly speaking, SOM^+^ INs fall into two morphological categories: Martinotti (cortical layers 2/3 and 5) and non-Martinotti cells (cortical layer 4 [188]) (Figure 4) [7].

### 8.1. Morphology of SOM^+^ INs and Expression of Neurochemical Markers

Martinotti cells display a variety of somatodendritic morphologies and are easily recognised by their translaminar axon with dense arborisations that are always directed towards cortical layer 1 (see Figure 1). It is because of this extensive axonal arborisation that they are suggested to act as gate keepers of neocortical activity [189]. Postsynaptic targets are the proximal and distal dendrites of pyramidal neurons [7,8,190,191], as well as neighbouring VIP^+^ and PV^+^ INs [192,193,194]. Within the somatosensory cortex, this type of SOM^+^ INs share significant electrical coupling [195,196].

Lastly, neurochemical profiles of SOM^+^ INs are complex. Immunocytochemical studies can identify six distinct and non-overlapping neurochemical subgroups in the cingulate cortex alone [3]. In fact, only a minority of SOM^+^ INs do not express another neuropeptide or calcium-binding protein. Most SOM^+^ INs express a specific neuropeptide (such as NPY) or calcium binding protein, with calretinin and/or calbindin being the most common. Furthermore, preprodynorphin and reelin identifies another neurochemical subgroup of SOM^+^ INs [178,197].

Non-Martinotti cells seem to represent the smaller fraction of the total population of SOM^+^ INs [4,30,198] and are a morphologically heterogenous group. Some are basket cells [169] that resemble fast-spiking PV^+^ INs [188]. Another subgroup consists of long-range GABAergic projection neurons [183,199,200,201]. These SOM^+^ IN subtypes often coexpress nNOS, NPY, the substance P receptor [199,200], or the neuronal nicotinic receptor modulator Lypd6 [202]. The function of these SOM^+^ projection neurons is unclear, yet one role appears to be auditory fear conditioning [203].

### 8.2. Electrophysiological Properties of SOM^+^ INs

Martinotti and non-Martinotti SOM^+^ INs exhibit distinct electrophysiological signatures [4,188]. Martinotti cells display a continuous action potential discharge pattern but some are also reported to show burst-spiking, stuttering, or rarely delayed firing patterns (Figure 1) [4,30]. Non-Martinotti cells in turn [188] resemble fast-spiking INs with regard to their single spike properties and passive membrane properties. Low-threshold calcium spikes are exhibited by a significant proportion of infragranular SOM^+^ INs [30,40,196] but only by a minority of supragranular SOM^+^ INs [4,25], which may be linked to cortical layer information processing.

## 9. PV^+^ and SOM^+^ Interneuron Connectivity

Granular and infragranular PV^+^ INs receive strong inputs from thalamic afferents and function as feed-forward inhibitors of pyramidal cells that likewise receive strong excitatory input from thalamic afferents, thereby enhancing the temporal fidelity of pyramidal cell responsiveness. Supragranular PV^+^ INs receive inputs from excitatory corticocortical afferents as well as from neighbouring pyramidal neurons (Figure 4) [149,204,205,206], indicating that PV^+^ INs not only provide feedforward but also feedback inhibition, preferentially targeting the proximal dendrites and the soma of pyramidal neurons. PV^+^ INs are known to innervate Chandelier cells, VIP^+^, CCK^+^, nNOS^+^, and SOM^+^ INs [194,207,208]. In contrast, SOM^+^ INs mostly receive excitatory inputs from neighbouring pyramidal neurons and weaker inputs from thalamic and corticocortical afferents, in turn preferentially inhibiting the distal and proximal dendrites of not only pyramidal cells but also PV^+^, nNOS^+^, and VIP^+^ INs (Figure 4) [192,193,208,209,210,211,212,213]. It is postulated that SOM^+^ INs are “ideally located within the cortical circuit to modulate sensory integration” [214] and provide not only lateral/feedback but also feedforward inhibition.

Activation of cortical afferents usually induces depressing responses in PV^+^ INs, whereas those onto SOM^+^ INs are typically facilitating [33,34,35,215,216,217]. The inhibitory input onto PV^+^ INs largely derives from SOM^+^, VIP^+^, and CCK^+^ INs. PV^+^ INs typically display electrical coupling with other PV^+^ INs and chandelier cells [194,218,219,220,221,222]. In contrast, SOM^+^ INs primarily receive inhibitory inputs from layer 1 INs, many of which are VIP^+^ INs [211]. Comparing the synaptic input frequency between supragranular PV^+^ and SOM^+^ INs, PV^+^ INs seem to receive excitatory and inhibitory synaptic inputs with a higher frequency (2–40 Hz vs. 1–10 Hz) [4,145,223,224,225,226,227,228]. The larger synaptic input onto PV^+^ INs in comparison to SOM^+^ INs might reflect their differential recruitment: it was recently shown that PV^+^ INs of the somatosensory cortex are preferentially recruited by long-range excitatory inputs [213].

In addition, the activity of both IN types is modulated by afferents from subcortical regions such as the nucleus raphé or the basal forebrain. Optogenetic stimulation of the latter strongly modulates the activity of prefrontal nNOS^+^/SOM^+^ INs and pharmacological activation of acetylcholine receptors has been shown to predominantly excite PV^+^ and SOM^+^ INs [229,230].

## 10. Functional Implications for PV^+^ and SOM^+^ INs

### 10.1. PV^+^ INs

From a functional perspective, neocortical PV^+^ INs are involved in maintaining cortical ‘up’ states, high gamma and ripple oscillations in the upper cortical layers, and in suppressing beta oscillations in the deeper cortical layers [140,231,232] during wakefulness. During sleep, cortical excitability is generally decreased [233,234] and PV^+^ INs show a distinct activity pattern across different sleep stages and may contribute to memory consolidation during sleep. In addition, while PV^+^ IN activity decreases during slow wave sleep (SWS), it increases during rapid eye movement (REM) sleep. Therefore PV^+^ INs are thought to provide increased cortical inhibition during REM sleep [235,236]. It has been further suggested that PV^+^ IN activity levels during wakefulness and sleep are accompanied by diurnal changes in the levels of perineuronal nets (PNNs) surrounding PV^+^ INs and by an increase in the PV protein itself [237]. Nevertheless, it would be beneficial to monitor both PNN and PV levels during different sleep stages and then correlate those directly to PV^+^ IN activity to really understand the functional significance of how PNNs may contribute to PV^+^ IN activity and excitability.

Selective inactivation of PV^+^ INs results in working memory impairments, cognitive deficits, and reduced behavioural flexibility and sociability in mice [238,239,240]. These data reflect a functional importance of PV^+^ INs for information processing.

### 10.2. SOM^+^ INs

Neocortical SOM^+^ INs are implicated in sound habituation, the reversal of sound habituation [241], stimulus-specific adaptation [242,243], centre-surround modulation [244], and fear conditioning [203]. In addition, a role for SOM^+^ INs in learning and memory and in higher cognitive function has been attributed: (1) activity of SOM^+^ INs regulates the spine density of pyramidal cells [167]; (2) SOM^+^ INs are preferentially active during the delay period of a working memory task [245]; (3) optogenetic activation of SOM^+^ INs suppresses the delay period and impairs behavioural performance [240]; (4) SOM^+^ INs are crucial for affective state discrimination [239]; and (5) social fear expression is dependent on activity of SOM^+^ INs and SOM^+^ inactivation reduces social fear behaviour [246,247]. It could be shown that gamma rhythm in the visual cortex (V1) is dependent on SOM^+^ (and PV^+^) INs activity [248]. Similarly, SOM^+^ projection neurons of the septo-hippocampal circuit play a role in the generation of rhythmic oscillations in the hippocampus [249,250,251] and the activity of SOM^+^ INs of the basal forebrain correlates with gamma band activity within the same cortex area [252]. SOM^+^ INs display a sleep stage-dependent activity profile and exhibit increased activity during SWS, suggesting that pyramidal cell excitability during SWS is mainly controlled by SOM^+^ INs [235].

### 10.3. Neurodevelopmental Disorders of PV^+^ and/or SOM^+^ IN Function

Behaviour is defined as “internally coordinated responses (actions or inactions) of whole living organisms (individuals or groups) to internal and/or external stimuli, excluding responses more easily understood as developmental changes” [253]. Every organism must thus be able to respond to external and internal stimuli in a context-dependent manner. Therefore, sensory information processing and integration must be stimulated or suppressed to induce a relevant and beneficial behavioural response. As mentioned earlier, GABAergic INs modulate the output signal of pyramidal neurons and help to heighten the contrast of processed information by different circuit motifs of inhibition. In doing so, GABAergic INs play an important role in suppressing or allowing sensory information processing to induce habituation or sensitisation to internal or external stimuli [238,239,240,241,242,243,244]. As such, it is not surprising that alterations in PV^+^ or SOM^+^ IN numbers or cortical displacement of PV^+^ and/or SOM^+^ INs are accompanied by unphysiological behavioural responses in animals and human patients that can manifest as mood disorders, working memory disorders, autism spectrum disorders, bipolar disorders, or cognitive impairments [254,255,256,257,258,259,260,261,262,263]. In addition, many of these disorders are accompanied by a higher incidence of epileptic seizures in animals and/or human patients [92,264,265,266].

## 11. Conclusions

The assembly of neural circuits is a multi-level process that is initiated by the birth of neurons and is accomplished by their correct positioning and wiring within a given cortical circuit. Adjustments to neural circuits occur continuously throughout life as do behavioural adaptations to experience. Proper network function necessitates that the correct number and types of neurons communicate with each other in a context-dependent manner. On a general scale, this means that a precise number of excitatory and inhibitory neurons are recruited to maintain a physiological network activity level. This means that excess numbers of neurons must be eliminated from the circuit and that this recruitment must also consider the subtype of the neuron that is being incorporated into a given neural circuit. Cortical INs are composed of diverse groups of cells, many of which are in turn made up of subgroups and further subordinates. These groups differ in their morphological, electrophysiological, neurochemical, and functional properties. It appears that subtype specificity in cortical INs is primarily organised by lineage but neuronal activity can have an impact on the IN phenotype. However, the details of how PV^+^ and/or SOM^+^ INs differentiate from the MGE during their development into the final phenotype, and whether subgroups of INs play a functional role, are only beginning to be understood.

## Data Availability

Not applicable.
